# Ultrasound-Assisted Versus Standard Catheter-Directed Thrombolysis for Acute Pulmonary Embolism: Insights From National Inpatient Sample

**DOI:** 10.1016/j.jscai.2024.101360

**Published:** 2024-04-05

**Authors:** Islam Shatla, Mahmoud El Iskandarani, Muhammad Zia Khan, Ahmed Elkaryoni, Ayman Elbadawi, Sachin S. Goel, Marwan Saad, Sudarshan Balla, Amir Darki, Islam Y. Elgendy

**Affiliations:** a Department of Internal Medicine, Kansas University Medical Center, Kansas City, Kansas; b Department of Internal Medicine, Eastern Connecticut Health Network, Manchester, Connecticut; c Division of Cardiovascular Disease, West Virginia University Heart and Vascular Institute, Morgantown, West Virginia; d Lifespan Cardiovascular Institute and Division of Cardiovascular Medicine, Warren Alpert Medical School of Brown University, Providence, Rhode Island; e Division of Cardiovascular Medicine, University of Texas Southwestern Medical Center, Dallas, Texas; f Division of Cardiovascular Medicine, Houston Methodist DeBakey Heart & Vascular Center, Houston, Texas; g Division of Cardiovascular Medicine, Loyola University Medical Center, Maywood, Illinois; h Division of Cardiovascular Medicine, Gill Heart Institute, University of Kentucky, Lexington, Kentucky

**Keywords:** catheter-directed thrombolysis, pulmonary embolism, ultrasound-assisted thrombolysis

## Abstract

**Background:**

Pulmonary embolism is one of the leading causes of morbidity and mortality in the United States. Catheter-directed therapies have emerged as a promising treatment for managing intermediate- and high-risk patients; however, data comparing standard catheter-directed thrombolysis (SCDT) and ultrasound-assisted thrombolysis (USAT) are limited. This study aimed to investigate trends, outcomes, and predictors of mortality of both modalities from a nationally representative sample.

**Methods:**

This analysis used data from the National Inpatient Sample years 2016-2020. The primary outcome was in-hospital mortality. A multivariable regression model was used to compare the outcomes.

**Results:**

Among 39,430 patients who received catheter-directed thrombolysis, 26,710 (76.8%) received SCDT and 8060 (23.2%) received USAT. The utilization of SCDT and USAT increased during the study years except for 2020. In-hospital mortality was lower among patients who received USAT (2.7% vs 3.8%; *P* = .04) compared with patients who received SCDT in the unadjusted analysis. On multivariable regression analysis, there was no difference in the incidence of in-hospital mortality between USAT and SCDT (odds ratio, 0.75; 95% CI, 0.52-1.08; *P* = .13). There were no significant differences between SCDT and USAT groups in the rate of bleeding adverse events including intracranial hemorrhage (0.6% vs 0.4%; *P* = .47), and nonintracranial major bleeding (4.2% vs 4.1%; *P* = .72).

**Conclusions:**

Ultrasound-assisted thrombolysis was associated with similar in-hospital mortality and bleeding complications compared with SCDT for acute pulmonary embolism. Further studies are warranted to confirm evaluate the long-term outcomes with both modalities.

## Introduction

Acute pulmonary embolism (PE) is a major cause of morbidity and mortality with over 100,000 deaths per year in the United States.^1^ Despite advances in the treatment of PE, the prognosis for intermediate- and high-risk patients has seen little improvement over the past 2 decades.^2^^,^^3^ Although anticoagulation therapy remains the cornerstone of therapy for most PE, reperfusion therapy could be considered among patients at high risk of decompensation.^4^^,^^5^ Social guidelines recommend systemic thrombolysis as the main reperfusion therapy for high-risk PE,^5^ which is associated with significant risk of intracranial bleeding reaching up to 1.5%.^6^

In recent years, catheter-directed therapies have emerged as a promising treatment for managing selected patients with intermediate- and high-risk PE.^4^ Currently, there are 2 techniques for catheter-directed thrombolysis (CDT): standard catheter-directed thrombolysis (SCDT) and ultrasound-assisted thrombolysis (USAT). SCDT involves the insertion of a side-hole catheter into the pulmonary artery with slow infusion of a low-dose thrombolytic agent directly into the thrombus. USAT combines the low-dose infusion of a thrombolytic agent with an ultrasound-equipped catheter system (EKOS Corp), which delivers high-frequency (2.2 MHz), low-power (0.5 W per element) acoustic energy.^7^ Although ultrasound does not dissolve the thrombus, it can facilitate the dissociation of fibrin strands, thereby improving the penetration of the thrombolytic agent.^8^^,^^9^ The current evidence comparing these 2 techniques is scarce. One randomized clinical trial and a few small retrospective studies^10^, ^11^, ^12^ evaluated surrogate outcomes (eg, hemodynamic parameters) and showed comparable findings with either modality. To better address this knowledge gap, we aimed to compare the in-hospital outcomes with USAT versus SCDT among patients with PE from a contemporary nationally representative dataset.

## Methods

### Data source

Data from the National Inpatient Sample (NIS) years 2016-2020 were used for this study. The NIS is sponsored by the Agency for Healthcare Research and Quality.^13^ The NIS includes hospital information for >7 million hospital discharges annually, accounting for 20% of all discharges from nonfederal hospitals in all 50 states. It also provides discharge weights that are used for computation of disease outcomes and health care utilization. Institutional review board approval and informed consent were not required for this study owing to the deidentified nature of the dataset.

### Study population

Patients with PE who received CDT from January 2016 through December 2020 were identified using the International Classification of Diseases, Tenth Revision (ICD-10), Clinical Modification (ICD-10 Procedure Coding System), code 3E06317. We excluded patients who underwent catheter-directed embolectomy (ICD-10 codes: 02CP3ZZ, 02CQ3ZZ, and 02CR3ZZ) and surgical embolectomy (ICD-10 codes: 02CP0ZZ, 02CQ0ZZ, and 02CR0ZZ) and those who received systemic thrombolytics (ICD-10 code 3E03317) during the index admission. We also excluded patients with diagnosis of acute stroke and acute limb ischemia because these conditions could be treated with CDT. We also excluded pregnancy-related PE.^14^^,^^15^ The study cohort was divided into 2 groups based on the use of ultrasound-guided intervention: SCDT vs USAT (ICD-10 codes: 6A750Z5, 6A750Z6, 6A750Z7, 6A750ZZ, 6A751Z5, 6A751Z6, 6A751Z7, and 6A751ZZ).

### Outcomes

The primary outcome was in-hospital mortality. Secondary outcomes included intracranial hemorrhage (ICH), nonintracranial bleeding (including hemothorax, hemopericardium, hemoperitoneum, gastrointestinal bleeding, hematuria, hematoma, and procedure-related bleeding), bleeding requiring transfusion, and health care resource utilization, including length of stay (LOS), hospitalization cost, discharge disposition, and predictors of mortality for the study cohort. For computing hospitalization costs, the cost-to-charge ratio files supplied by Healthcare Cost and Utilization Project (HCUP) were applied to the total hospital charges.

### Statistical analysis

All analyses were conducted using the appropriate weighting, stratifying, and clustering samples following HCUP regulations. Data are presented as mean ± SD for continuous variables and number (percentage) for categorical variables. The 2 groups (SCDT vs USAT) were compared using Student *t* test for continuous and χ^2^ for categorical variables. Trend analysis was performed using a linear regression model. In-hospital mortality, ICH, and nonintracranial bleeding were compared using a multivariable regression model. Predictors of mortality for the study cohort were also analyzed in multivariate regression model as well. The covariates selected for adjustment included age, sex, hypertension, diabetes mellitus, saddle PE, cardiogenic shock, vasopressor use, coronary artery disease, heart failure, atrial fibrillation, peripheral vascular disease, end-stage renal disease, chronic obstructive pulmonary disease, peptic ulcer disease, coagulopathy, obesity, smoking, hospital bed size, and teaching status. A 2-tailed *P* value of .05 was used for significance testing, and an odds ratio (OR) with 95% confidence interval (CI) was used as a measure of effect size reported by logistic regression. A sensitivity analysis was conducted by excluding hospitalizations in 2020 to eliminate the potential effect of COVID-19 pandemic. A falsification end point analysis was conducted to assess residual confounders after performing regression model. For this analysis, we evaluated other outcomes that are not pathophysiologically related to acute PE or CDT (ie, acute liver failure). All analyses were performed using SPSS Statistics 24.0 (IBM Corp). For data elements with less than 11 observations, we reported (<11) to ensure compliance with HCUP recommendations.

## Results

**Figure 1:**
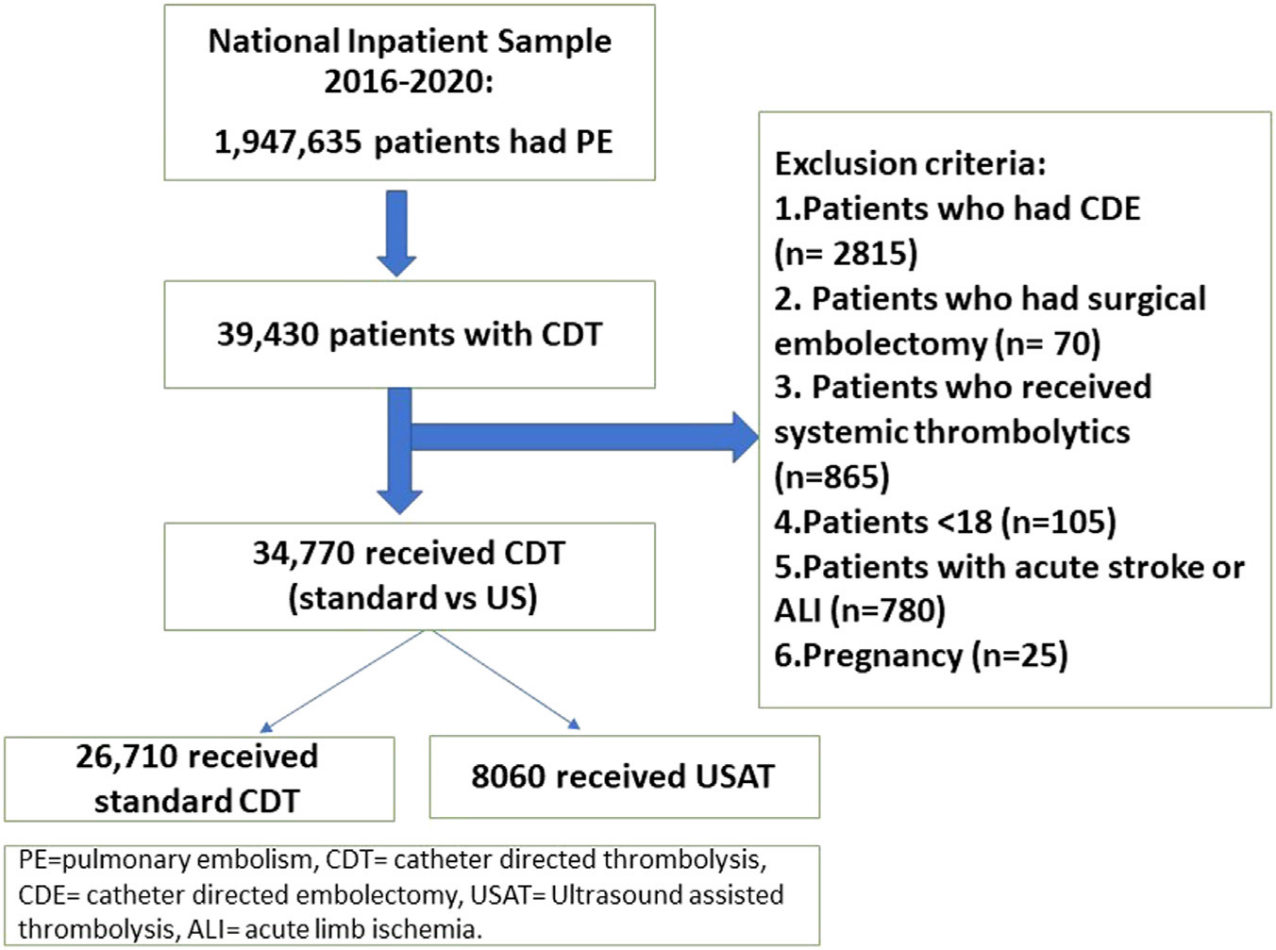
**Flow chart for patient selection.** ALI, acute limb ischemia; CDE, catheter-directed embolectomy; CDT, catheter-directed thrombolysis; PE, pulmonary embolism; US, ultrasound; USAT, ultrasound-assisted thrombolysis.

**Figure 2:**
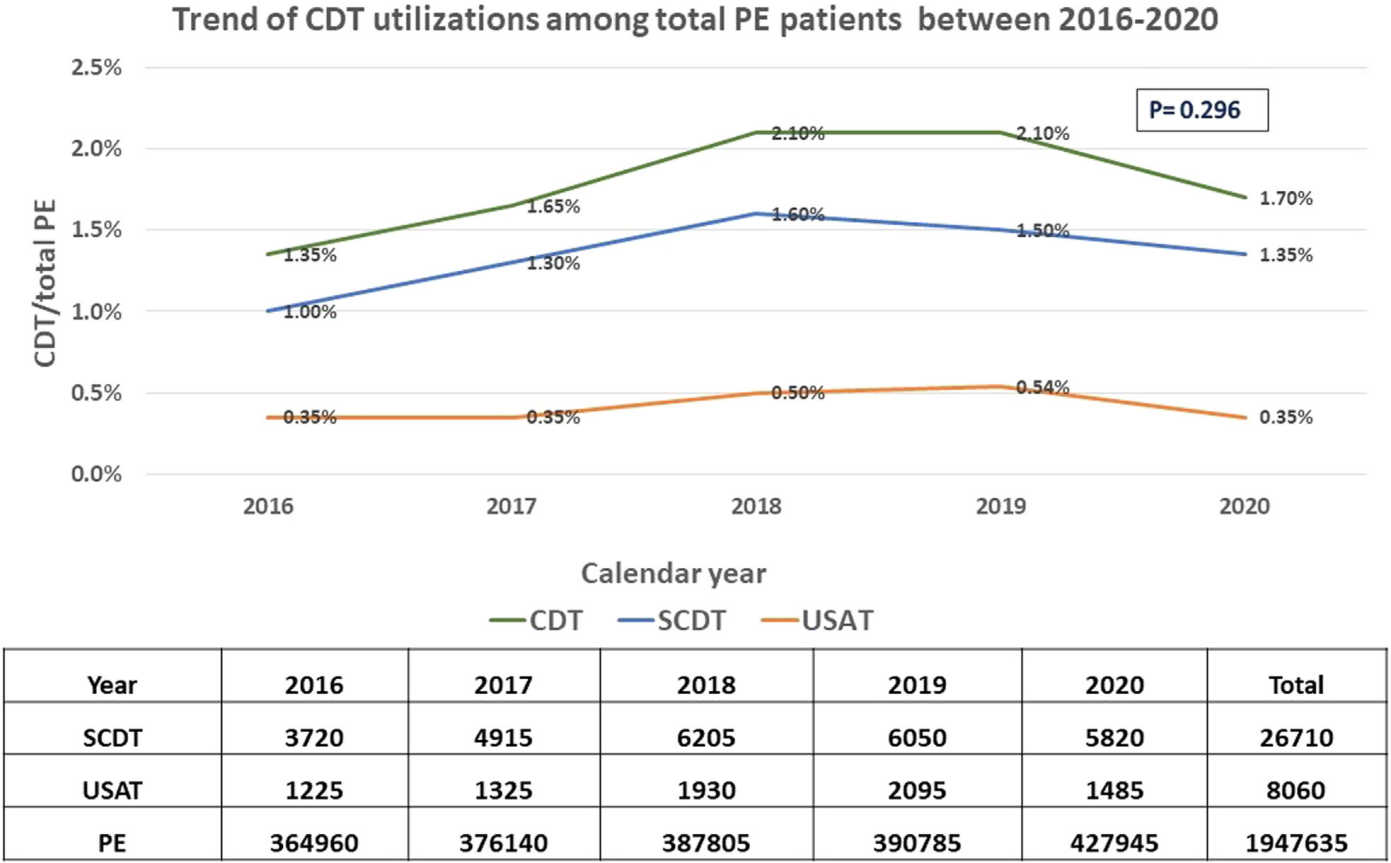
**Trend of utilization of standard catheter-directed thrombolysis (SCDT) and ultrasound-assisted thrombolysis (USAT) between 2016 and 2020.** CDT, catheter-directed thrombolysis; PE, pulmonary embolism.

The study population included 39,430 patients who received CDT (Figure 1); of which, 26,710 (76.8%) received standard CDT and 8060 (23.2%) received USAT. The mean age for SCDT group was 60.5 ± 14.7 years vs 60.7 ± 14.6 years in the USAT group. The utilization of SCDT and USAT increased during the study years except for 2020 as shown in Figure 2. The baseline characteristics of the study population stratified based on the use of SCDT vs USAT are reported in Table 1. Patients who underwent SCDT were more likely to have coagulopathy and liver disease, whereas those who received USAT were more likely to have coronary artery disease. Cardiogenic shock, vasopressor use, mechanical ventilation use, mechanical circulatory support use, and saddle PE were comparable in both groups. Patients receiving CDT were likely treated in large and teaching hospitals.

**Table 1:** Baseline characteristics.

Variables	SCDT (n = 26,710)	USAT (n = 8060)	*P* value
Age, y	60.5 ± 14.7	60.7 ± 14.6	.27
Female sex	12,405 (46.5)	3910 (48.5)	.14
Race			.18
White	19,105 (74.0)	5705 (73.0)	
Black	4640 (18.0)	1510 (9.3)	
Hispanic	1355 (5.2)	350 (4.5)	
Asian or Pacific Islander	195 (0.8)	50 (0.6)	
Native American	100 (0.4)	<10 (0.1)	
Other	430 (1.7)	185 (2.4)	
Diabetes mellitus	3180 (11.9)	1020 (12.7)	.43
Hypertension	16,765 (62.8)	5180 (64.3)	.27
Atrial fibrillation	2870 (10.7)	830 (10.3)	.61
Cardiac arrhythmias	6390 (23.9)	1960 (24.3)	.75
HF	4595 (17.20)	1320 (16.4)	.45
CAD	3070 (11.5)	1070 (13.3)	.06
PVD	830 (3.1)	290 (3.6)	.31
DVT	15,500 (58.0)	4620 (57.3)	.62
Coagulopathy	4580 (17.1)	1215 (15.1)	.06
Any form of anemia	4950 (18.5)	1585 (19.7)	.33
Deficiency anemias	1215 (4.5)	400 (5.0)	.49
Peptic ulcers	100 (0.4)	30 (0.4)	.99
ESRD	140 (0.5)	40 (0.5)	.90
Liver disease	1,310 (4.9)	300 (3.7)	.05
COPD	5250 (19.7)	1545 (19.2)	.66
Alcohol use disorder	25 (0.1)	20 (0.2)	.13
Smoking status	3330 (12.5)	1060 (13.2)	.47
Obesity	10,885 (40.8)	3375 (41.9)	.44
Hospital bed size			.38
Small	4465 (16.7)	1275 (15.8)	
Medium	7680 (28.8)	2500 (31.0)	
Large	1455 (54.5)	3375 (41.9)	
Hospital status			.01
Rural	1010 (3.8)	475 (5.9)	
Urban nonteaching	4965 (18.6)	1645 (20.4)	
Urban teaching	20,735 (77.6)	5940 (73.7)	
Hospital category			.13
Government, nonfederal	2445 (9.2)	575 (7.1)	
Private, not-for-profit	20,900 (78.2)	6445 (80)	
Private, investor-owned	3365 (12.6)	1040 (12.9)	
Primary payer			.88
Medicare	11,875 (44.5)	3565 (44.3)	
Medicaid	2525 (9.5)	690 (8.6)	
Private insurance	10,010 (37.5)	3090 (38.4)	
Self-pay	1290 (4.8)	390 (4.8)	
No charge	100 (0.4)	40 (0.5)	
Other	890 (3.3)	275 (3.4)	
Saddle PE	9785 (36.6)	2890 (35.9)	.58
Vasopressors use	400 (1.5)	85 (1.1)	.18
Cardiogenic shock	1070 (4.0)	255 (3.2)	.11
Mechanical ventilation	2575 (9.6)	700 (8.7)	.25
ECMO	125 (0.5)	20 (0.2)	.24

Values are mean ± SD or n (%).

CAD, coronary artery disease; COPD, chronic obstructive pulmonary disease; DVT, deep venous thrombosis; ECMO, extracorporeal membrane oxygenation; ESRD, end-stage renal disease; HF, heart failure; PE, pulmonary embolism; PVD, peripheral vascular disease; SCDT, standard catheter-directed thrombolysis; USAT, ultrasound-assisted thrombolysis.

### In-hospital outcomes

**Figure 3:**
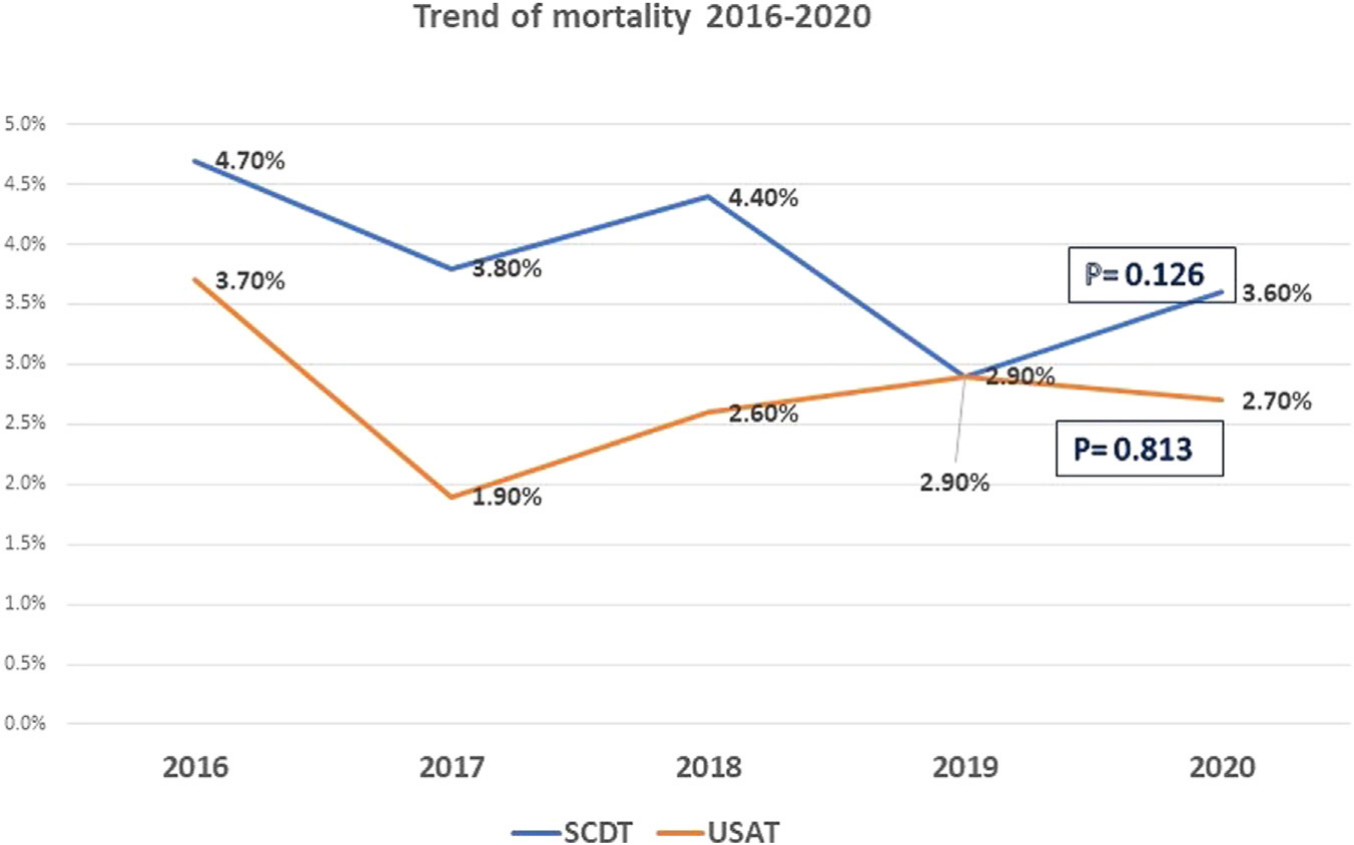
**Trend of mortality among patients who received standard catheter-directed thrombolysis and ultrasound-assisted thrombolysis between 2016 and 2020**. SCDT, standard catheter-directed thrombolysis; USAT, ultrasound-assisted thrombolysis.

In-hospital adverse events and complications in both groups are listed in Table 2. In-hospital mortality was lower among patients who received USAT (2.7% vs 3.8%; *P* = .04) compared with patients who received SCDT. In-hospital mortality rates did not change in both groups during the study period as shown in Figure 3. The sensitivity analysis excluding 2020 was largely consistent (2.7% vs 3.9%; *P* = .06). There were no significant differences between SCDT and USAT groups in the rate of bleeding adverse events including ICH (0.6% vs 0.4%; *P* = .31), nonintracranial bleeding (4.2% vs 4.1%; *P* = .85), procedure-related bleeding (0.2% vs 0.1%; *P* = .37), and bleeding requiring transfusion (3.2% vs 2.4%; *P* = .08), respectively.

**Table 2:** In-hospital outcomes.

Outcome	SCDT (n = 26,710)	USAT (n = 8060)	*P* value
Mortality	1020 (3.8)	220 (2.7)	.04
Intracranial hemorrhage	155 (0.6)	30 (0.4)	.31
Nonintracranial hemorrhage	1100 (4.1)	340 (4.2)	.85
Procedural bleeding	65 (0.2)	<11 (0.1)	.37
Blood transfusion	855 (3.2)	190 (2.4)	.08

Values are n (%).

SCDT, standard catheter-directed thrombolysis; USAT, ultrasound-assisted thrombolysis.

USAT was associated with less health care utilization as evidenced by shorter median LOS (IQR) (4 [3-6] days vs 4 [3-7] days; *P* < .001), a higher proportion of patients discharged to home (70.3% vs 68.7%; *P* = .04), and lower median cost (IQR) ($90,915 [67,753-134,120] vs $94,290 [68,043-139,374]; *P* = .01) (Table 3).

**Table 3:** Resource utilization.

Variables	SCDT (n = 26,710)	USAT (n = 8060)	*P* value
Length of stay, d	4 (3-7)	4 (3-6)	<.001
Total charges, $	94,290 (68,027-139,406)	90,915 (67,704-134,147)	.01
Disposition			.04
Routine	18,345 (68.7)	5670 (70.3)	
Short-term hospital	395 (1.5)	120 (1.5)	
Another facility	3480 (13.0)	925 (11.5)	
Home health care	3360 (12.6)	1105 (13.7)	
Against medical advice	105 (0.4)	15 (0.2)	
Died	1020 (3.8)	220 (2.7)	

Values are median (IQR) or n (%).

SCDT, standard catheter-directed thrombolysis; USAT, ultrasound-assisted thrombolysis.

### Multivariate regression analysis

**Figure 4:**
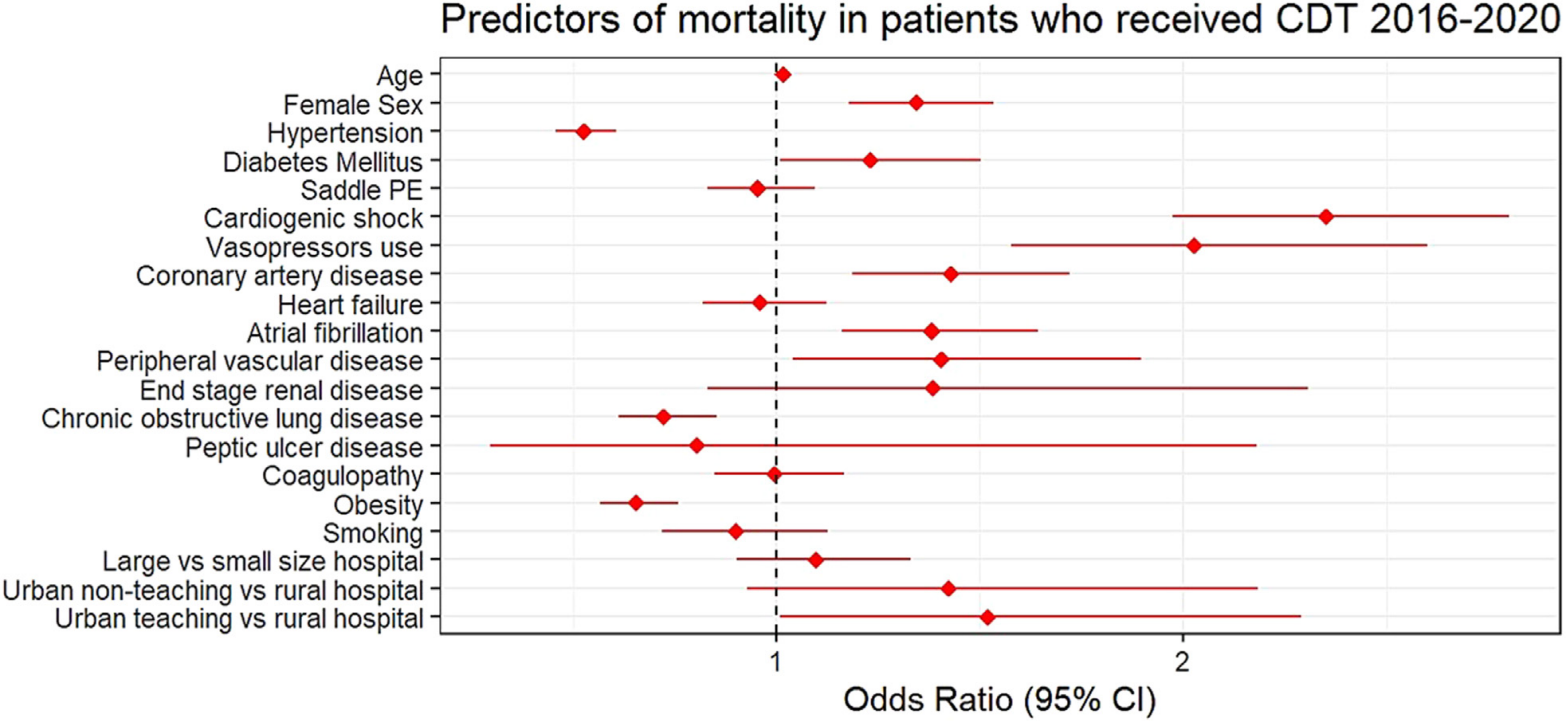
**Factors associated with mortality among patients who received catheter-directed thrombolysis (CDT) (standard CDT and ultrasound-assisted thrombolysis).** PE, pulmonary embolism.

On multivariable regression analysis, there was no difference in the incidence of in-hospital mortality between USAT and SCDT (OR, 0.75; 95% CI, 0.52-1.08; *P* = .13) (Central Illustration). Additionally, there was no difference between both groups in ICH (OR, 0.72; 95% CI, 0.30-1.75; *P* = .47) and nonintracranial bleeding (OR, 0.78; 95% CI, 0.21-2.95; *P* = .72) (Table 4). On multivariate analysis, predictors of mortality among the study cohort included cardiogenic shock (OR, 2.35; 95% CI, 1.97-2.80; *P* < .001), vasopressor use (OR, 2.0; 95% CI, 1.57-2.60; *P* < .001), and advancing age (OR, 1.01; 95% CI, 1.01-1.02; *P* < .001) (Figure 4). The falsification end point analysis showed that there was no difference in the rate of acute liver failure (adjusted OR, 1.41; 95% CI, 0.76-2.58; *P* = .27) between USAT and SCDT.

**Central Illustration:**
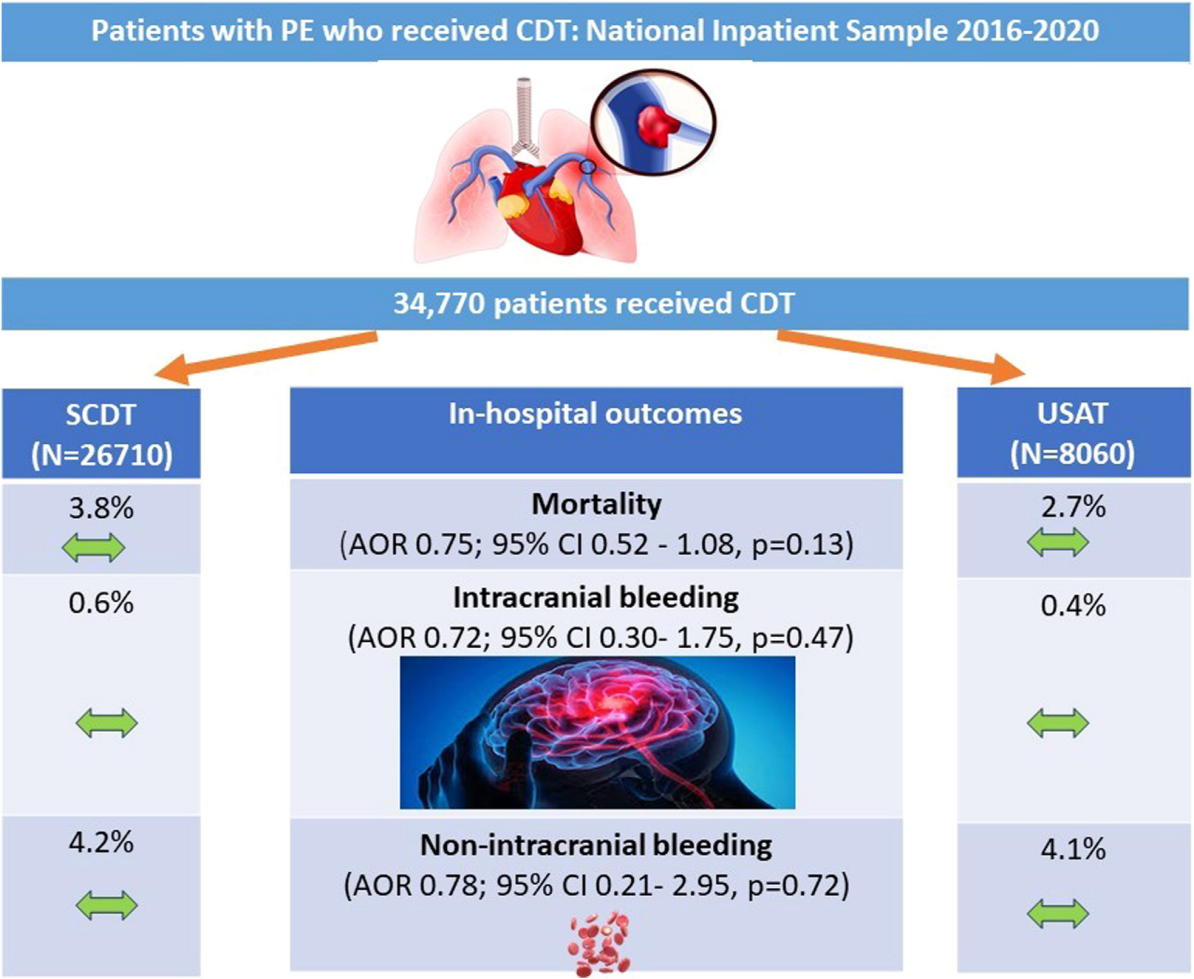
In-hospital outcomes with standard catheter-directed thrombolysis (SCDT) vs ultrasound-assisted thrombolysis (USAT) for acute PE. AOR, adjusted odds ratio; CDT, catheter-directed thrombolysis; PE, pulmonary embolism.

**Table 4:** Unadjusted and adjusted odds ratio of clinical outcomes.

Outcome	Odds ratio (95% CI)	*P* value
Mortality (unadjusted)	0.71 (0.50-0.99)	.04
Mortality (adjusted)	0.75 (0.52-1.08)	.13
Intracranial hemorrhage (unadjusted)	0.64 (0.26-1.52)	.32
Intracranial hemorrhage (adjusted)	0.72 (0.30-1.75)	.47
Nonintracranial hemorrhage (unadjusted)	0.69 (0.19-2.48)	.57
Nonintracranial hemorrhage (adjusted)	0.78 (0.21-2.95)	.72

Variables used for adjustment: age in years at admission, indicator of sex, hypertension, diabetes mellitus, cardiogenic shock, ventilator use short and long term, vasopressor use, saddle pulmonary embolism, coronary artery disease, congestive heart failure, atrial fibrillation, peripheral vascular disease, end-stage renal disease, chronic obstructive pulmonary disease, peptic ulcer disease, coagulopathy, obesity, and smoking status.

## Discussion

In this nationwide contemporary observational analysis, we compared the outcomes with USAT versus SCDT for acute PE. The main findings were as follows: (1) there was no difference in the adjusted in-hospital mortality between both modalities; (2) there was no difference in the adjusted rates of ICH and non-ICH major bleeding events between both groups; (3) USAT was associated with lower resource utilization, that is, shorter LOS, lower cost, and higher likelihood of discharge to home.

Multiple retrospective studies have been conducted to compare USAT with SCDT. However, these studies had a small sample size ranging from 20 to 98 patients with intermediate- and high-risk PE.^11^^,^^12^^,^^16^^,^^17^ These studies focused on surrogate hemodynamic parameters. The only randomized clinical trial, SUNSET sPE (Standard vs Ultrasound-Assisted Catheter Thrombolysis for Submassive Pulmonary Embolism), compared the outcomes of 81 patients with intermediate-risk PE who underwent USAT or SCDT.^10^ The study demonstrated no difference in pulmonary thrombotic load score.^10^ However, it showed that patients who received SCDT had shorter LOS and smaller right ventricle-to-left ventricle (RV/LV) ratio compared with those for patients who received USAT.^10^ It should be noted that the study was not powered to detect these end points and was limited by the small sample size and lack of standardized tissue plasminogen activator dosing regimen. A meta-analysis of these studies including the SUNSET sPE (Standard vs Ultrasound-Assisted Catheter Thrombolysis for Submassive Pulmonary Embolism) showed similar effects on thrombotic load score (modified Miller score).^18^ A greater reduction in RV/LV ratio was seen in the SCDT group but it showed a similar mortality rate and bleeding rate in both groups.^18^

A prior analysis of the National Readmission Database for the year 2016 including 2060 patients showed that there was no difference in the incidence of in-hospital mortality between USAT and SCDT.^19^ In our analysis of NIS database from 2016 to 2020, we present contemporary real-world data for patients who received CDT representing the largest sample size, and there was no difference in the rates of in-hospital mortality with both therapies. The use of USAT and SCDT in these years trended upward except for the year 2020 when the utilization of both procedures decreased. This reduction could be attributed to COVID-19 pandemic. It is important to note that the overall in-hospital mortality rate was low in this analysis because we excluded patients who received systemic thrombolysis and other advanced therapies. Although it has been postulated that USAT would reduce the dose and duration of the thrombolytic agents and subsequently reduce the risk of bleeding, the rates of ICH were very low with both modalities. This suggests that SCDT is safe and has a similar safety profile when compared with USAT. Our study demonstrated that there was no difference in bleeding outcomes between both the USAT and SCDT groups, which is consistent with prior comparative studies.^11^^,^^12^^,^^16^^,^^18^^,^^20^

In our analysis, LOS was shorter in the USAT group than in the SCDT group. We postulate that this might be related to the reduction in the duration of the infusion of thrombolytic therapy. Previous studies showed conflicting results with mostly similar LOS between the 2 procedures,^12^^,^^17^^,^^21^ whereas other studies demonstrated shorter hospital stay for LOS in the SCDT as opposed to our findings.^10^^,^^18^ The reduction in LOS in the USAT group translated into a reduction in hospital charge compared with that in the SCDT group.

The findings of our study should be interpreted in the context of some limitations. First, data on the infusion dose and duration for the thrombolytic agent are unavailable in this database. In addition, the database lacks data on the concomitant anticoagulation. Second, information on the hemodynamic parameters before and after the procedure is lacking. Specifically, there is no information regarding the changes in RV/LV ratio or computed tomography angiography–based assessment of residual thrombus (modified Miller index). Other risk-stratification tools of patients based on the established PE-risk categories or any longer-term end points (readmission rates, rates of chronic thromboembolic pulmonary hypertension, and PE-specific mortality) similarly could not be assessed. Third, given the administrative nature of the NIS, it is susceptible to coding errors. Finally, this study is observational and retrospective in nature, there is a possibility of selection bias. We performed adjusted and falsification end point analyses to mitigate this risk.

## Conclusion

In this contemporary nationwide observational analysis of hospitalizations with PE, USAT was associated with similar in-hospital mortality and bleeding complications compared with SCDT. Although our results suggest that both modalities are associated with comparable in-hospital outcomes, further studies are warranted to evaluate long-term outcomes.
